# Impact of Saharan Dust and *SERPINA1* Gene Variants on Bacterial/Fungal Balance in Asthma Patients

**DOI:** 10.3390/ijms26052158

**Published:** 2025-02-27

**Authors:** Ainhoa Escuela-Escobar, Javier Perez-Garcia, Elena Martín-González, Cristina González Martín, José M. Hernández-Pérez, Ruperto González Pérez, Inmaculada Sánchez Machín, Paloma Poza Guedes, Elena Mederos-Luis, María Pino-Yanes, Fabian Lorenzo-Díaz, Mario A. González Carracedo, José A. Pérez Pérez

**Affiliations:** 1 Instituto Universitario de Enfermedades Tropicales y Salud Pública de Canarias (IUETSPC), Universidad de La Laguna (ULL), 38200 San Cristóbal de La Laguna, Spain; aescuela@ull.edu.es (A.E.-E.); cgonzama@ull.edu.es (C.G.M.); florenzo@ull.edu.es (F.L.-D.); joanpere@ull.edu.es (J.A.P.P.); 2 Genomics and Health Group, Department of Biochemistry, Microbiology, Cell Biology and Genetics, Universidad de La Laguna (ULL), 38200 San Cristóbal de La Laguna, Spain; jpegarci@ull.edu.es (J.P.-G.); emarting@ull.edu.es (E.M.-G.); mdelpino@ull.edu.es (M.P.-Y.); 3 Pulmonology Unit, Hospital Universitario N. S. de Candelaria (HUNSC), 38010 Santa Cruz de Tenerife, Spain; jmherper@hotmail.com; 4 Allergy Department, Complejo Hospitalario Universitario de Canarias (HUC), 38320 San Cristóbal de La Laguna, Spain; glezruperto@gmail.com (R.G.P.); zerupean67@gmail.com (I.S.M.); pozagdes@hotmail.com (P.P.G.); elenamederosluis@gmail.com (E.M.-L.); 5 CIBER de Enfermedades Respiratorias, Instituto de Salud Carlos III, 28029 Madrid, Spain; 6 Instituto de Tecnologías Biomédicas (ITB), Universidad de La Laguna (ULL), 38200 San Cristóbal de La Laguna, Spain

**Keywords:** asthma, microbial DNA concentration, Saharan dust intrusions, *SERPINA1*, SNV, *16S rRNA*, *18S rRNA*

## Abstract

The Canary Islands, a region with high asthma prevalence, are frequently exposed to Saharan Dust Intrusions (SDIs), as are a wide range of countries in Europe. Alpha-1 antitrypsin (*SERPINA1* gene) regulates the airway’s inflammatory response. This study analyzed the combined effect of SDI exposure and *SERPINA1* variants on bacterial/fungal DNA concentrations in saliva and pharyngeal samples from asthmatic patients. Bacterial and fungal DNAs were quantified by qPCR in 211 asthmatic patients (GEMAS study), grouped based on their exposure to daily PM_10_ concentrations. Associations between SDI exposure, microbial DNA concentrations, and nine variants in *SERPINA1* were tested using linear regression models adjusted for confounders. The ratio between bacterial and fungal DNA was similar in saliva and pharyngeal samples. SDI exposure for 1–3 days was enough to observe significant microbial DNA change. Increased bacterial DNA concentration was detected when SDI exposure occurred 4–10 days prior to sampling, while exposure between days 1 and 3 led to a reduction in the fungal DNA concentration. The T-allele of *SERPINA1* SNV rs2854254 prevented the increase in the bacterial/fungal DNA ratio in pharyngeal samples after SDI exposure. The bacterial/fungal DNA ratio represents a potential tool to monitor changes in the microbiome of asthmatic patients.

## 1. Introduction

Exposure to Saharan Dust Intrusions (SDI), a key component of the environmental exposome [1], has been associated with respiratory health risks, mainly due to its inflammatory and oxidative effects on the respiratory system [2]. The Canary Islands are situated about 100 km northwest of the African coast [3], at the western end of the so-called “dust belt” [4], being exposed to SDI throughout the year [5]. Transport of Saharan dust to the Canary Islands occurs through two primary mechanisms, depending on the season. During the winter, dust is carried by low-level continental African trade winds (Harmattan winds) deflected northwestward by Atlantic cyclones. In contrast, during summer, dust is mainly transported to the Canary Islands by the northern branch of the high-altitude Saharan Air Layer (SAL) [5]. This situation makes this region particularly relevant for studying the impact of SDI on respiratory health. However, SDI not only affects the Canary Islands, but it also extended across continental Europe, with increasing frequency and intensity in recent years. This trend has been linked to climate change, which is enhancing the frequency and extent of these Saharan dust events [6]. Saharan dust is primarily composed of minerals but also carries different biological materials, including bacteria, fungi, viruses, and pollen spores, which can survive long-distance transport [7]. Therefore, SDI modifies the microbiome profiles of the atmosphere, introducing desert soil-associated bacteria to distant ecosystems [8,9,10], and even carrying microorganisms across oceanic barriers [11]. However, the effects of SDI exposure on the abundance and diversity of the respiratory microbiome and the potential exposure to air-transported pathogens remain an emerging research area [11,12].

Air quality during SDI is influenced by different factors, such as PM_10_ concentration levels, chemical composition, or particle size distribution, among others [13]. Notably, SDI in the Canary Islands usually exceeds the safety limits described in the European Directive 2008/50/EC, which sets daily PM_10_ concentration to a maximum of 50 µg/m^3^. For instance, in February 2020, unprecedented levels of airborne particles were recorded, exceeding 1000 µg/m³ in some areas [14]. Dust particles can penetrate the airways differently according to their aerodynamic diameter. Airborne particles with a diameter ≤ 10 µm (PM_10_) mostly reach the upper airways, leading to their inflammation [15,16,17]. It is well known that the airway microbiome plays a crucial role in maintaining respiratory health. Moreover, the disruptions in its balance, known as dysbiosis, have been associated with common airway diseases such as asthma [11,18,19]. Therefore, studying whether the inflammatory effects caused by exposure to PM_10_ modulate the respiratory microbiome is of great interest, especially in regions continuously exposed to SDI, such as the Canary Islands.

According to the Global Initiative for Asthma, asthma is a heterogeneous respiratory disease characterized by chronic airway inflammation, which includes different respiratory symptoms [20]. This disease affects about 300 million people worldwide, causing around 1000 deaths per day [20]. Asthma prevalence in the Canary Islands is higher than in other areas of Spain, representing approximately 18% of the population [21], and is also highly prevalent worldwide [22]. It has been shown that various genetic and environmental risk factors influence asthma development and severity [23]. Among them, specific variants of the *SERPINA1* gene have been recently associated with asthma exacerbations, both in the Canary Islands [24] and other populations [25].

The *SERPINA1* gene encodes Alpha-1 antitrypsin (AAT), a glycoprotein that belongs to the serine protease inhibitor superfamily. The main function of AAT is to protect body tissues, particularly the lungs, from damage caused by proteolytic enzymes such as neutrophil elastase [26]. Recently, important immunomodulatory properties have been described for this protein [27]. Genetic variants in the *SERPINA1* gene can affect both the production and functionality of AAT, which could lead to AAT deficiency (AATD) [28]. The most-studied deficient variants are Pi*Z (rs28929474) and Pi*S (rs17580) alleles [28]. Both alleles, but especially Pi*Z, cause significant reductions in circulating AAT levels [29], increasing the risk of developing chronic pulmonary and liver diseases [28]. However, more than 500 *SERPINA1* variants have been described in this gene, of which probably more than 160 have implications for AATD [30].

Since AAT plays a crucial role in modulating inflammation, individuals with unbalanced *SERPINA1* gene expression may exhibit an unregulated inflammatory environment in their airways, which in turn could promote the colonization and persistence of certain respiratory pathogens [31,32,33,34]. Overall, the inflammatory effect caused by SDI exposure, together with the deregulated inflammatory response caused by specific *SERPINA1* variants, could alter the microbiota abundance and composition, driving progression and exacerbations of respiratory diseases, such as asthma. In the present work, we evaluated how SDI exposure, along with variants of the *SERPINA1* gene, may influence the concentration of microbial DNA in saliva and pharyngeal samples of asthmatic patients.

## 2. Results

### 2.1. Microbial DNA Concentrations in the Study Population

This study included 211 asthma patients (Table S1) with a median age of 46.2 years (66.8% female) and a median asthma onset age of 18 years. Subjects had a median FEV_1_ and FVC of 86.9 and 91.6 (% predicted), respectively. Immunological markers showed median IgE levels of 140.3 UI/mL and eosinophil counts of 300 cells/µL. ICS use reached 95.3% of patients, while 26.5% of them used systemic corticosteroids, and 42.2% reported asthma exacerbations in the last 6 months. Considering treatment steps according to GINA 2020, severe asthma was predominant (92.4%), with a T2 asthma phenotype present in 92.0% of individuals. However, 42.3% of patients showed well-controlled asthma according to the asthma control questionnaire (ACQ) score. Only 14.7% received biological therapy, and 31.5% reported antibiotic use.

**Figure 1:**
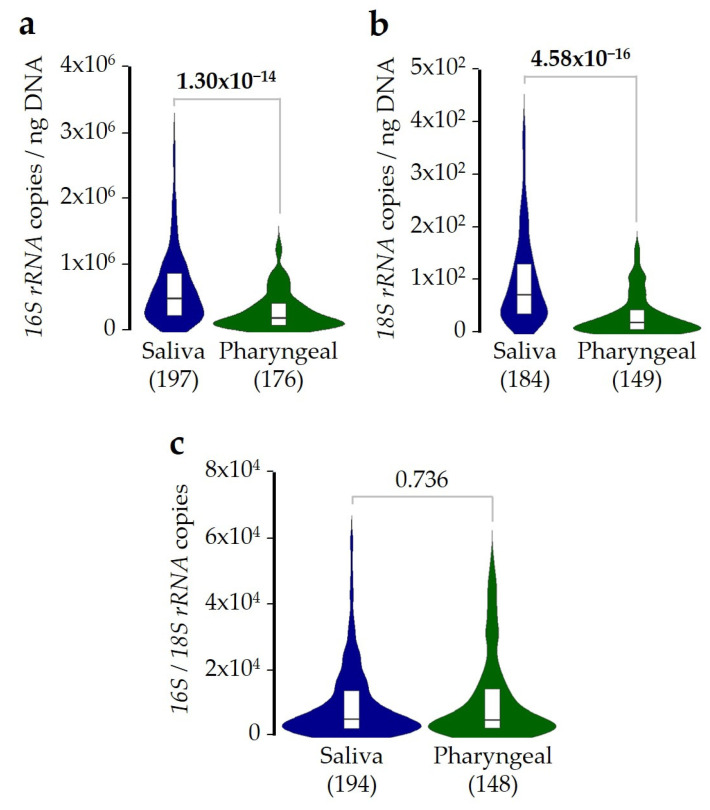
Bacterial, fungal, and ratio of bacterial/fungal DNA concentrations in saliva and pharyngeal samples. DNA purified from saliva (blue) or pharyngeal (green) samples was used for real-time qPCR measurement of DNA concentrations of (**a**) bacteria, (**b**) fungi, or (**c**) the bacterial/fungal ratio. In all cases, median and interquartile ranges are shown. The number of samples, after removal of outliers, is indicated below each plot. Differences were evaluated using the Mann–Whitney U test. Statistically significant *p*-values (<0.05) are highlighted in bold.

The copy number of the *16S rRNA* gene per nanogram of purified DNA was significantly higher in saliva than in pharyngeal samples (median of 4.6 × 10^5^ and 1.8 × 10^5^ copies, respectively) (Figure 1a), and the same was observed for the *18S rRNA* gene (median of 70.4 and 22.8 copies) (Figure 1b). However, the ratio between concentrations of the target DNA sequences from bacteria and fungi was very similar in saliva and pharyngeal samples (median of 4487.5 and 4365.9, respectively) (Figure 1c).

When demographic and clinical variables listed in Table S1 were tested for their associations with bacterial, fungal, and bacterial/fungal ratios of DNA concentrations (Table S2), age, sex, recruiting island, and use of antibiotics were identified as potential confounding variables and were included as covariates in the multiple linear regression models onwards. Nominal associations were found for asthma control with fungal and bacterial DNA concentrations in saliva and pharyngeal samples, respectively. Moreover, the asthma T2 phenotype was nominally associated with the fungal DNA concentration in saliva and with the bacterial/fungal DNA concentration ratio in pharyngeal samples. However, these associations were not supported after correction for multiple comparisons.

Interestingly, differences in microbial DNA concentrations previously detected between saliva and pharyngeal samples (Figure 1) remained statistically supported by corrected models, and after correction for multiple comparisons, no differences were found for the ratio of bacterial/fungal DNA concentrations between the two sample types (Table S3). Overall, these results indicate that bacterial and fungal DNA concentrations are about 2.5 and 3.1 times higher in saliva than in pharyngeal samples, respectively. However, although bacterial DNA was about four thousand times higher than fungal in both sample types, the balance of DNA concentrations did not differ between them.

### 2.2. Bacterial/Fungal DNA Ratio Is Associated with SDI Exposure

**Figure 2:**
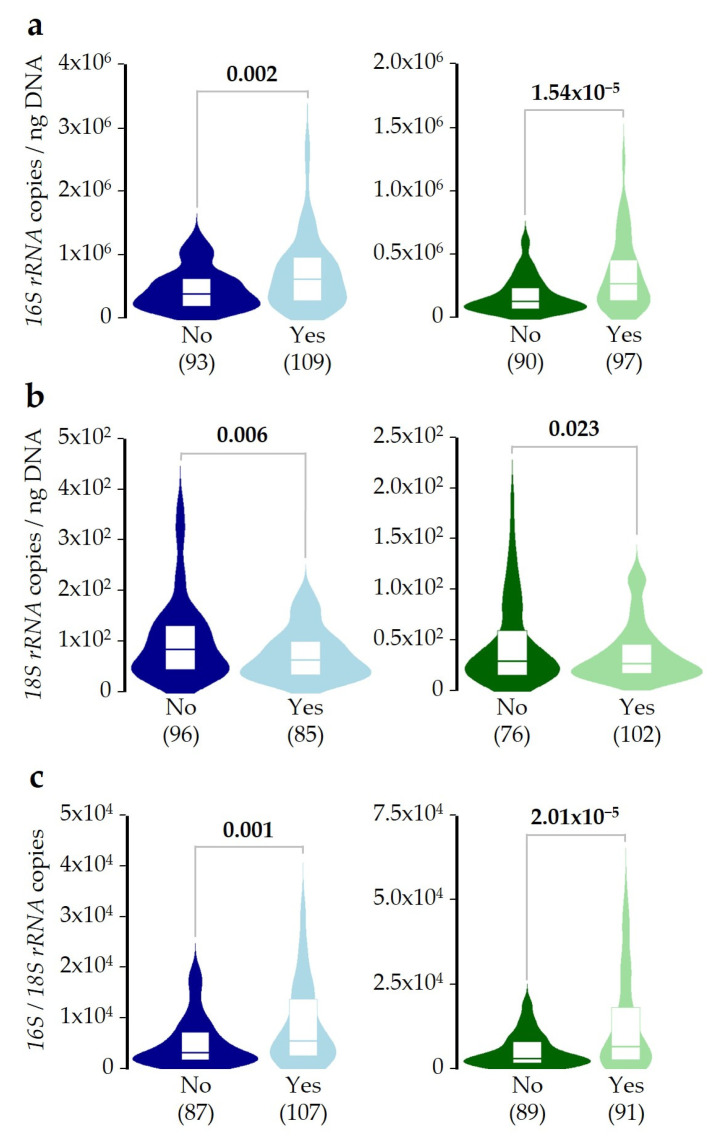
Effect of SDI exposure on bacterial, fungal, and ratio of bacterial/fungal DNA concentrations. Patients were subdivided considering whether they were exposed (Yes) or not (No) to an SDI in the 10-day period prior to sampling. (**a**) Bacterial DNA concentration in saliva (blue) or pharyngeal (green) samples. (**b**) Results for fungal DNA concentration. (**c**) Results for the ratio of bacterial/fungal DNA concentrations. In all cases, the Mann–Whitney U test was used to test differences between groups. Significant *p*-values (<0.05) are highlighted in bold. The sample numbers after the removal of outliers are shown below each plot.

To evaluate the influence of SDI in the microbial DNA concentrations, patients were distributed as Not-exposed (considering the 10-day period before sampling) or Exposed (at least one day of exposure in the same period). Results showed that exposed patients showed higher bacterial DNA concentration in both saliva and pharyngeal samples when compared with the Not-exposed group (Figure 2a). On the other hand, fungal DNA concentration was lower in both sample types of the Exposed group (Figure 2b). Furthermore, when the ratio of bacterial/fungal DNA concentrations was studied, differences between both patient groups were confirmed. This result reveals that the analysis of the balance between bacterial and fungal DNA concentrations offers a greater sensitivity to monitor changes in microbiota in both sample types (Figure 2c). These results were confirmed by regression models adjusted by age, sex, recruiting island, use of antibiotics (last 2 months), and PCs, and after correction for multiple comparisons (Table S4).

**Figure 3:**
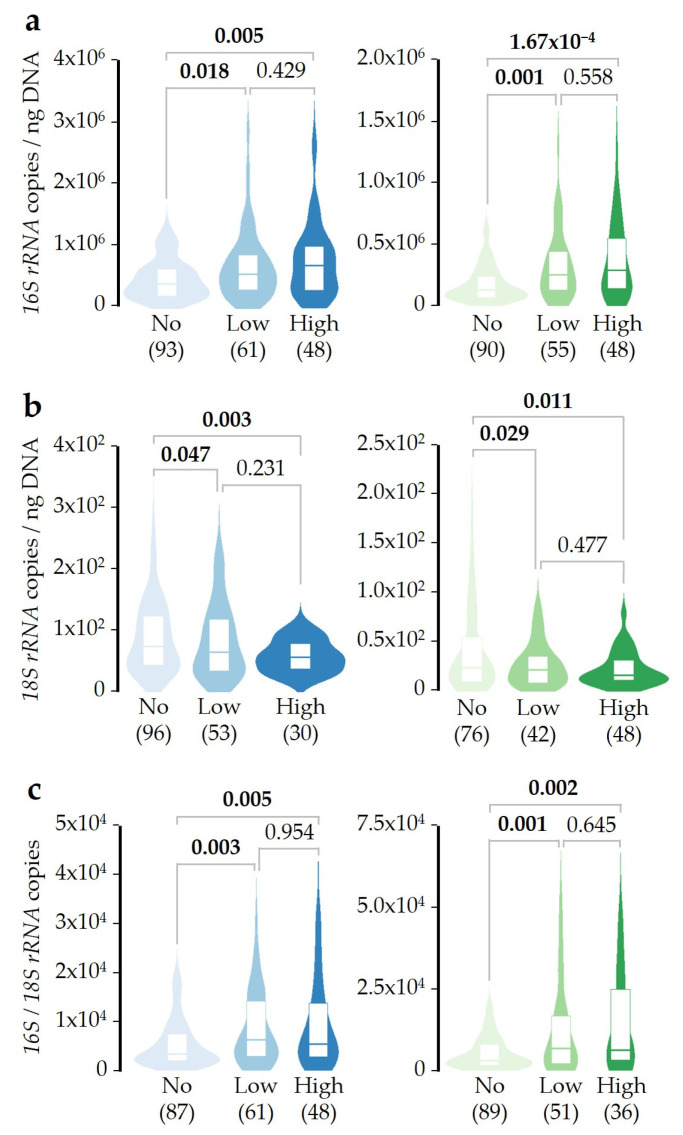
Non-accumulative effect of SDI exposure over bacterial, fungal, and ratio of bacterial/fungal DNA concentrations. (**a**) Bacterial DNA concentration was measured in saliva (blue) or pharyngeal (green) samples. Patients were distributed as Not-exposed (0 out of 10 days prior to sampling; light), Low-exposed (1–3 days; medium), or High-exposed (4–6 days; dark). (**b**) Results obtained as in A, but for the fungal *18S rRNA* gene copy number. (**c**) Results obtained as in A, but for the ratio of bacterial/fungal DNA concentrations. In all cases, the Mann–Whitney U test was used to evaluate all possible pairwise comparisons between DNA concentrations. Significant *p*-values (<0.05) are highlighted in bold, and the sample size after outlier removal from each group is shown below each plot.

To study if SDI exposure changes microbial DNA concentrations in an accumulative manner, patients were distributed into Not-exposed (0 days of exposure during the 10 days prior to sampling), Low-exposed (1–3 days of exposure), or High-exposed (4–6 days of exposure) groups. Both Low- and High-exposed patient groups showed a higher bacterial DNA concentration in saliva and pharyngeal samples when compared with the Not-exposed group (Figure 3a). Conversely, fungal DNA concentration was lower in both sample types, at a similar level after low- or high-exposure to SDI (Figure 3b). When the ratio between bacterial and fungal DNA concentrations was studied, these differences were clearer (Figure 3c). These results were confirmed by adjusted linear regression models (Table S5). Since no statistically significant differences were detected between Low- and High-exposed groups, once patients were low-exposed to SDI, a greater number of exposure days seems not to produce a drastic effect on the balance between both groups of microorganisms.

### 2.3. SDI Exposure Time-Course Effect over Microbial DNA Concentrations

**Figure 4:**
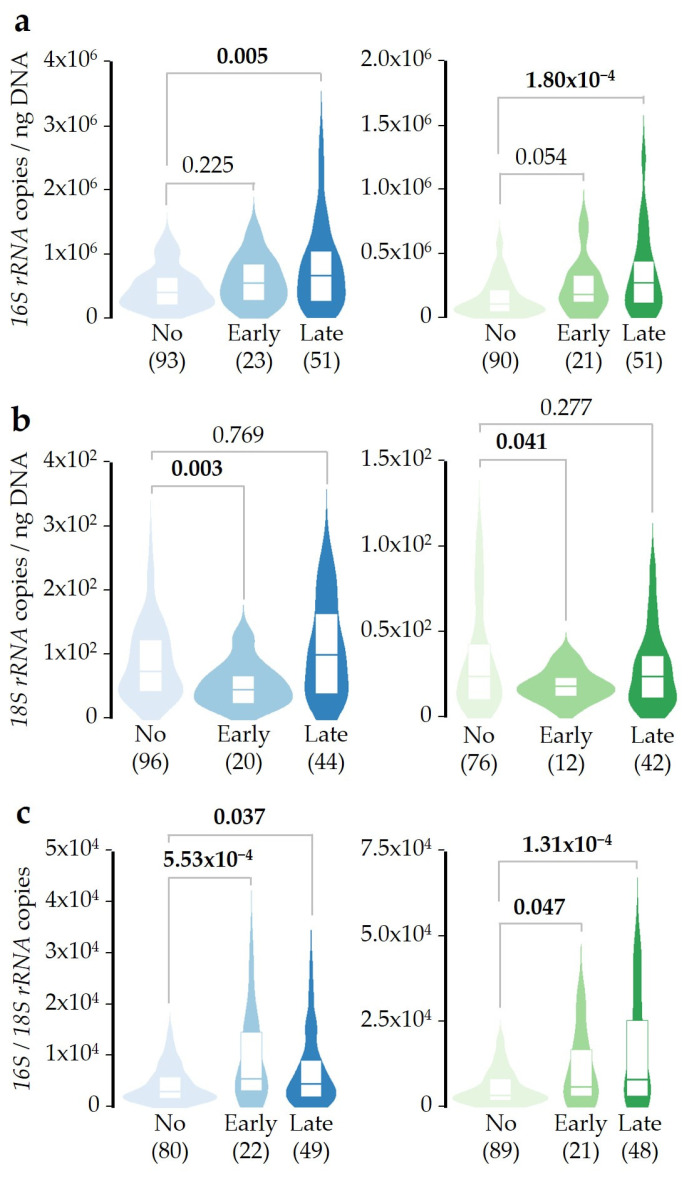
Time-course effect of SDI exposure over bacterial, fungal, and ratio of bacterial/fungal DNA concentrations. (**a**) Bacterial DNA concentration was measured in saliva (blue) or pharyngeal (green) samples. Patients were distributed as Not-exposed (0 out of 10 days prior to sampling; light), Early-exposed (exposed between days 1 and 3 prior to sampling; medium), or Late-exposed (exposed between days 4 and 10 prior to sampling; dark). (**b**) Results obtained as in A, but for fungal *18S rRNA* gene copy number per nanogram of purified DNA. (**c**) Results obtained as in A, but for the ratio of bacterial/fungal DNA concentrations. In all cases, the Mann–Whitney U test was used to compare with the Not-exposed group. Significant *p*-values (<0.05) are highlighted in bold, and the sample size after outlier removal from each group is shown below each plot.

In order to study the kinetics of the changes in microbial DNA concentrations induced by SDI exposure, patients were distributed into three groups considering the period of time between SDI exposure and sampling. Groups were defined as Not-exposed (no exposure within the 10-day period prior to sampling), Early-exposed (when the exposure took place during days 1–3 before sampling, but not previously), or Late-exposed (when the exposure occurred at days 4–10 prior to sampling). Results showed higher bacterial DNA concentration in both saliva and pharyngeal samples when patients were late-exposed to SDI. However, no statistically significant differences were observed for the Early-exposed group (Figure 4a). On the other hand, early-exposed patients showed lower fungal DNA concentrations in saliva and pharyngeal than the Not-exposed group, but these differences were not detected when the Late-exposed group was evaluated (Figure 4b). Therefore, fungal DNA concentration in saliva and pharyngeal samples seems to change faster than bacteria.

When the balance between bacterial and fungal DNA concentrations was tested, both the Early- and Late-exposed groups showed higher ratios in saliva and pharyngeal samples than Not-exposed patients (Figure 4c). The early increase in the bacterial/fungal ratio of DNA concentrations after SDI exposure can be easily explained by the reduction in the fungal DNA concentration previously detected (Figure 4b), while its late increase is caused by the higher bacterial DNA concentration observed at this time interval (Figure 4a). As in previous analyses, results were confirmed by adjusted linear regression models and after correction for multiple comparisons (Table S6). These results confirm that the bacterial/fungal ratio of DNA concentrations is a more sensitive predictor of changes in microbiome abundance, since modifications can be detected in both time periods.

### 2.4. Associations of SERPINA1 Gene Variants with Microbial DNA Concentrations

*SERPINA1* genotypes for a set of nine Single Nucleotide Variants (SNVs) were recovered from genome-wide genotyping data [24]. The copy number of their minor alleles was tested for associations with bacterial, fungal, and the ratio of bacterial/fungal DNA concentrations in both sample types, using adjusted linear regression models (Table S7).

Interestingly, the presence of the T-allele of the rs2854254 SNV was accompanied by a lower bacterial DNA concentration and a higher fungal DNA concentration in pharyngeal samples. These correlations showed nominal significance (*p* = 0.046 and *p* = 0.053, respectively) when separately analyzed. However, the association between the T-allele and a lower ratio of bacterial/fungal DNA concentrations was strongly supported after Bonferroni correction (*p* = 2.01 × 10^−3^; Table S7).

**Figure 5:**
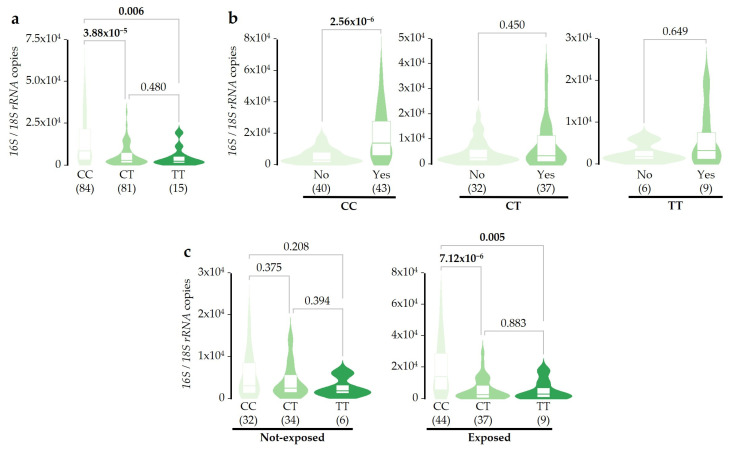
Effect of SNV rs2854254 and SDI exposure over the ratio of bacterial/fungal DNA concentrations in pharyngeal samples. (**a**) Patients were distributed considering their rs2854254 genotype, and the pharyngeal ratio of bacterial/fungal DNA concentrations was compared. (**b**) The pharyngeal ratio of bacterial/fungal DNA concentrations was compared between exposed and not-exposed individuals inside each genotype group. (**c**) The pharyngeal ratio of bacterial/fungal DNA concentrations was compared between genotypes inside each exposure group. In all cases, the Mann–Whitney U test was used. Significant *p*-values (<0.05) are highlighted in bold, and the sample size after outlier removal from each group is shown below each plot.

The analysis of individual genotypes revealed that the T-allele of the rs2854254 SNV acts as a dominant genetic marker, since the mentioned ratios were not significantly different between CT and TT genotypes, but both were significantly lower than the ratio observed in the CC genotype (Figure 5a). To evaluate the possible effect of these SNVs on the microbial balance after SDI exposure, the ratio was compared inside each genotype group between Exposed or Not-exposed patients (Figure 5b). Results show that the increase in the ratio of bacterial/fungal DNA concentrations after SDI exposure previously observed (Figure 2c) was only replicated among patients that were homozygous for the rs2854254 C-allele (Figure 5b). Moreover, the higher ratio detected in patients with the CC genotype when the entire cohort was analyzed (Figure 5a) was due to the subset of patients exposed to SDI, since no differences were observed between genotype groups for Not-exposed patients (Figure 5c). Overall, these results show that allele T of rs2854254 could mitigate the disbalance between bacterial and fungal DNA concentrations detected in pharyngeal samples after SDI exposure. These results were confirmed by adjusted linear regression models and after correction for multiple comparisons (Table S8).

## 3. Discussion

As far as we know, this study is the first to demonstrate that SDI exposure can modify the microbial DNA concentration in saliva and pharyngeal samples from asthmatic patients. Initially, we found that bacterial and fungal DNA concentrations were about 2–3 times higher in saliva than in pharyngeal samples, while their ratio was essentially the same in the two sample types, with bacterial DNA concentrations about 4.4 × 10^3^ times higher than fungal. Next, we described how SDI exposure alters microbial balance, increasing and reducing the bacterial and fungal DNA concentrations, respectively (Figure 2). The fact that this disturbance was similar in magnitude in both sample types makes the results more robust. The effect of SDI exposure seems to be not additive, as patients exposed from 1 to 3 days showed a similar change in DNA concentrations as those who were exposed for more days (Figure 3). Moreover, the concentration of fungal DNA decreased faster after SDI exposure, while the concentration of bacterial DNA took longer to increase (Figure 4).

Several studies have confirmed that the concentration of airborne bacteria increases during dust events, suggesting potential respiratory health risks [35,36,37]. However, to date, no studies have specifically analyzed the effects of SDI exposure on microbial DNA concentration. This study is the first to demonstrate how bacterial and fungal DNA concentrations in saliva and pharyngeal samples are altered following exposure to SDI. However, it must be considered as a limitation of the present study the relatively small sample size, only focused on asthmatic patients. Therefore, the conclusions might not reflect the genetic and environmental interactions in individuals without pre-existing respiratory conditions or with other diseases. Additionally, due to the sample availability, this study was conducted under a case-control design. However, future longitudinal studies would be valuable to obtain a deeper understanding of the temporal distribution of the observed changes in microbial DNA concentrations. Nevertheless, the presented findings provide new insights into the microbiological impact of SDI on the upper respiratory tract and open the door to addressing future studies focused on elucidating the molecular pathways probably involved in the inflammatory process that modulates these changes in asthmatic patients.

The number of microbes present in saliva may change by an order of magnitude as a result of daily perturbations, and another level of complexity in this context is the quantification of living microorganisms [38]. Therefore, accurate quantification of microbial load in saliva remains challenging, and different strategies have been developed [38,39]. It is necessary to highlight that our aim was not to estimate the microbial cell number (i.e., the microbial load), but to obtain a potential marker that can be easily measured by real-time qPCR with reproducible results and to evaluate its usefulness for monitoring the effect of SDI exposure on microbial DNA concentrations and bacterial/fungal balance in our set of asthmatic patients. In this sense, we implemented two strategies to standardize results. First, the concentration of total purified DNA was used for normalization of microbial DNA quantification instead of sample volume, which is more prone to experimental errors. As a limitation, a commercial DNA purification kit specially recommended for the isolation of bacterial DNA has been used in the present study [40], which could explain the low concentration of fungal DNA detected from both sample types. Nevertheless, this kit has been widely used for mycobiome studies, with reproducible results [41].

Secondly, we have introduced in our analysis the ratio of *16S/18S rRNA* gene copies. We consider this ratio to have a greater sensitivity and to be a more accurate predictor of changes in microbial DNA concentrations, since the influence of the variable proportions of recovered human/microbial DNA between samples is simply eliminated from the equation. The ratio could serve as a valuable tool for tracking microbial changes in response to external environmental factors, such as SDI, but further research is needed to validate its clinical utility. Additionally, the ratio is based on two independent PCRs from the same sample (each with their corresponding replicates) and not only monitors changes in bacteria but also in the fungal component of the microbiome. It is important to highlight that bacterial and fungal DNA concentrations were inferred from the copy number of *16S* or *18S rRNA* genes per nanogram of purified DNA, respectively. Nevertheless, this assumption is certainly limited by interspecific variations in the copy number of the target DNA sequences within microbial genomes, particularly among fungi [42]. In favor of this approach, the balance between both groups of microorganisms has been revealed as a key factor in the development of several diseases [43,44]. One important consideration of these results is how changes in DNA concentration are translated into the bacterial and fungal microbiome profiles. Consequently, future metagenomic studies should be addressed to solve this question.

Finally, we found that the balance between bacterial and fungal DNA concentrations is not altered after SDI exposure when patients carry a specific variant of the *SERPINA1* gene (rs2854254 T-allele) (Figure 5). Recently, it has been reported that this T-allele increases the *SERPINA1* mRNA splicing in whole blood [30]. Therefore, serum AAT that reaches pharyngeal mucosa could be increased in the presence of the rs2854254 T-allele, contributing to regulating the inflammatory response caused by SDI exposure. The increase in AAT serum levels in blood as a consequence of the T-allele could also enhance the AAT antibacterial properties [34] or even its ability to modulate the fungal colonization [32], thus reducing the imbalance of the bacterial/fungal DNA concentrations. These hypotheses should be tested in the future, opening the door to future research in this field. Moreover, other genetic factors influencing immune responses and microbial composition should be explored to find additional interactions that could contribute to changes in the amount of microbial DNA.

## 4. Materials and Methods

### 4.1. Patients Included in This Study

We studied a subset of 211 asthmatic patients from the Genomics and Metagenomics of Asthma Severity (GEMAS) study, recruited between January 2018 and March 2020 in Tenerife and La Palma (Canary Islands, Spain). The study was carried out according to the code of ethics of the World Medical Association (Declaration of Helsinki) and received ethical approval from the ethics committees of participant centers (approval 29/17; date 26 April 2017). A consent form for participation was distributed to all participants and signed [45]. Saliva samples and pharyngeal swabs were collected as described elsewhere [40]. Demographic and clinical data (Table S1) were recovered as age, sex, recruiting island, season, body mass index (BMI), age of asthma onset, FEV_1_ and FVC % predicted, FEV_1_/FVC, immunoglobulin E levels, eosinophil counts, use of antibiotics (last 2 months), use of biological therapy (last 6 months), use of inhaled corticosteroids (last 6 months), use of systemic corticosteroids (last 6 months), presence/absence of asthma exacerbations (last 6 months), and asthma control test results [45].

### 4.2. Definition of Saharan Dust Intrusions

Data about air quality parameters were retrieved from the Air Quality Control and Surveillance Network of the Canary Islands [46]. Daily concentration (µg/m^3^) of particulate matters with a diameter ≤ 10 µm (PM_10_) was established as the main variable to define groups of patients, as PM_10_ values are heavily correlated with the presence of SDI in this region [4]. Daily values of PM_10_ concentrations were obtained from environmental stations placed on Tenerife (n = 27) and La Palma (n = 4) islands, from January 2018 to March 2020, and the average was calculated. Days with SDI were established when average PM_10_ concentrations reached at least 50 µg/m^3^, according to the EU air quality standards [47], and then confirmed using the retrospective natural events reports, annually published by the Spanish government [48]. The control group included asthma patients who had not been exposed to SDI within the 10 days before sampling. Details of PM_10_ concentrations are shown in Table S9 and summarized in Figure S1.

### 4.3. Bacterial and Fungal DNA Quantification

DNA was purified from saliva samples and pharyngeal swabs using the Pathogen Lysis Tubes S (Qiagen, Hilden, Germany) and the QIAamp UCP Pathogen Mini kit (Qiagen, Hilden, Germany) [40]. The concentration of the purified DNA was measured by fluorimetry. Standardized TaqMan-based quantitative PCR (qPCR) assays were used to measure the copy number of bacterial *16S rRNA* [49] or fungal *18S rRNA* [50] genes. Each qPCR, with a final volume of 10 µL, included 0.05–20 ng of DNA template, each amplification primer at 1.8 µM, 1X LightCycler 480 Probes Master (Roche, Basel, Switzerland), and one TaqMan probe at 0.2 µM. The qPCR conditions included an initial denaturation step (95 °C; 10 min), followed by 45 cycles of denaturation (95 °C; 10 s), annealing–extension (60 °C; 60 s), and fluorescence capture (72 °C; 1 s), using a LightCycler 480 Instrument II (Roche, Basel, Switzerland). All samples were tested by at least three technical replicates.

The quantification cycle (Cq) value for each technical replicate was obtained by the second derivative method. Technical replicates were retained only when their Cq differed in less than 0.5 cycles, and qPCR plates were validated when the mean Cq obtained for the negative control replicates was higher than 35 cycles. Samples with a mean Cq that differed in less than three cycles with respect to the negative control were considered negatives. Fluorescence data were loaded into the DART-PCR 1.0 software [51], and amplification efficiency was obtained for each sample.

The absolute number of *16S–18S rRNA* gene copies was calculated by interpolation of the mean Cq value for each sample in their respective calibration curves, which were prepared from serial dilutions of the ZymoBIOMICS Microbial Community DNA Standard (Zymo Research, Irvine, CA, USA). Those samples with an amplification efficiency out of the interval mean ± 1.96 standard deviation, defined by the standards of the calibration curve, were excluded from this analysis. Finally, the abundance of bacterial or fungal DNA in saliva and pharyngeal exudates was assumed to be mirrored by the copy number of *16S* or *18S rRNA* genes per nanogram of purified DNA, respectively.

### 4.4. Statistical Analysis

Data analysis was performed using RStudio v4.2.3 [52]. Outliers were independently inspected using the interquartile range (IQR) criterion (1.5× IQR above the third quartile or below the first quartile) for each group and excluded before each analysis. Distributions of bacterial and fungal DNA concentrations were tested for normality using the Kolmogorov–Smirnov test (n > 50). The Mann–Whitney U test was applied to compare independent variables without normal distribution. Multiple linear regression models were performed to compare bacterial, fungal, or the ratio of bacterial/fungal DNA concentrations per nanogram of purified DNA, both in saliva and pharyngeal samples, after a log transformation of data to obtain a normal distribution (confirmed as stated above). Associations were also tested with allele-additive models (presence of 0, 1, or 2 copies of the assessed allele) for 9 Single Nucleotide Variants (SNVs) from the *SERPINA1* gene, which were previously genotyped using the Infinium Global Screening Array-24 kit v3.0 (Illumina, San Diego, CA, USA) for the same set of subjects [24]. All models were adjusted by age, sex, recruiting island, use of antibiotics (last 2 months), and genetic ancestry (principal components [PCs] of genome-wide genotype data) [45]. Statistical significance was declared based on a *p*-value < 0.05 and a False Discovery Rate (FDR) < 0.05 after multiple comparison correction using the *p.adjust* function [53].

## 5. Conclusions

In summary, our findings reveal that exposure of asthmatic patients to SDI alters the balance between bacterial and fungal DNA in their saliva and pharynx. This environmental perturbation can be accurately monitored by measuring the ratio between the copy number of *16S rRNA* and *18S rRNA* genes per nanogram of purified DNA. Exposure to SDI leads to a decrease in fungal DNA concentration after 24–72 h, while bacterial DNA concentration subsequently increases in a non-accumulative manner. Additionally, the rs2854254 T-allele of the *SERPINA1* gene counteracts the SDI-induced disturbance of microbial balance.

## Data Availability

The datasets analyzed during the current study are available upon reasonable request to the corresponding author.

## Supplementary Materials

The following supporting information can be downloaded at: https://www.mdpi.com/article/10.3390/ijms26052158/s1.
