# Supplementary material for: Impact of Saharan Dust and SERPINA1 Gene Variants on Bacterial/Fungal Balance in Asthma Patients
Source: Int J Mol Sci. 2025 Feb 27;26(5):2158. doi: 10.3390/ijms26052158 (PMC11899813; doi:10.3390/ijms26052158)
Supplement: Supplementary file 1 [file ijms-26-02158-s001.zip › ijms-3456544-supplementary.pdf]

Supplementary Materials

Impact of Saharan Dust and *SERPINA1* Gene Variants on Bacterial/Fungal Balance in Asthma Patients

Ainhoa Escuela-Escobar [1,2], Javier Perez-Garcia [2], Elena Martín-González [2], Cristina González Martín [1], José M. Hernández-Pérez [3], Ruperto González Pérez [4], Inmaculada Sánchez Machín [4], Paloma Poza Guedes [4], Elena Mederos-Luis [4], María Pino-Yanes [2,5,6], Fabian Lorenzo-Díaz [1,2], Mario A. González Carracedo [1,2,*] and José A. Pérez Pérez [1,2]

1 Instituto Universitario de Enfermedades Tropicales y Salud Pública de Canarias (IUETSPC), Universidad de La Laguna (ULL), 38200 San Cristóbal de La Laguna, Spain; aescuela@ull.edu.es (A.E.-E.); cgonzama@ull.edu.es (C.G.M.); florenzo@ull.edu.es (F.L.-D.); joanpere@ull.edu.es (J.A.P.P.)
2 Genomics and Health Group, Department of Biochemistry, Microbiology, Cell Biology and Genetics, Universidad de La Laguna (ULL), 38200 San Cristóbal de La Laguna, Spain; jpegarci@ull.edu.es (J.P.-G.); emarting@ull.edu.es (E.M.-G.); mdelpino@ull.edu.es (M.P.-Y.)
3 Pulmonology Unit, Hospital Universitario N. S. de Candelaria (HUNSC), 38010 Santa Cruz de Tenerife, Spain; jmherper@hotmail.com
4 Allergy Department, Complejo Hospitalario Universitario de Canarias (HUC), 38320 San Cristóbal de La Laguna, Spain; glezruperto@gmail.com (R.G.P.); zerupean67@gmail.com (I.S.M.); pozagdes@hotmail.com (P.P.G.); elenamederosluis@gmail.com (E.M.-L.)
5 CIBER de Enfermedades Respiratorias, Instituto de Salud Carlos III, 28029 Madrid, Spain
6 Instituto de Tecnologías Biomédicas (ITB), Universidad de La Laguna (ULL), 38200 San Cristóbal de La Laguna, Spain
* Correspondence: mgonzalc@ull.edu.es

.

Supplementary Tables

Table S1. Clinical and demographic characteristics of the asthmatic patients included in this study.

Variable type	Variable		N	Asthma patients[a]
**Demographic**	Age (years)		211	46.2 (31.5 - 62.0)
	Sex (females), N (%)		211	141 (66.8)
	Recruiting island (Tenerife), N (%)		211	99 (46.9)
	Recruiting season (Winter), N (%)		211	154 (73.0)
**Clinical**	BMI		188	28.0 (25.0 - 32.9)
	Age of asthma onset (years)		189	18 (6 - 40)
	FEV_1 (% predicted)		200	86.9 (72.7 - 95.9)
	FVC (% predicted)		199	91.6 (80.5 - 100.8)
	FEV_1/FVC (% predicted)		199	76.8 (77.4 - 83.5)
	IgE levels (UI/ml)		191	140.3 (39.7 - 459.2)
	Eosinophil counts (cells/µl)		198	300 (100 - 500)
	Use of antibiotics, N (%)		210	67 (31.5)
	Biological therapy*, N (%)		211	31 (14.7)
	ICS use*, N (%)		211	201 (95.3)
	Systemic corticosteroids use*, N (%)		211	56 (26.5)
**Asthma features**	Exacerbations*, N (%)		211	89 (42.2)
	Asthma control, N (%)	Well controlled	194	82 (42.3)
		Partially controlled	194	57 (29.4)
		Poorly controlled	194	55 (28.4)
	Asthma severity, N (%)	Mild	197	2 (1.0)
		Moderate	197	11 (5.6)
		Severe	197	184 (92.4)
	T2 asthma, N (%)		201	185 (92.0)

[a]Continuous variables (age, asthma age onset, BMI, FEV_1, FVC, FEV_1/FVC, IgE levels, and eosinophil counts) were summarized with the median and interquartile range (in brackets). Categorical variables (sex, recruiting island, season, use of antibiotics (last 2 months), biological therapy, exacerbations, ICS use, systemic corticosteroids use, asthma control, asthma severity, and T2 asthma) were summarized as counts for each group and percentages (in brackets). Predicted values of lung function measurements were estimated using the Global Lung Function Initiative (GLI) 2012 equations. Asthma exacerbations were defined by the use of oral corticosteroids, emergency room visits, and/or asthma-related hospitalizations. Asthma control was assessed by the asthma control questionnaire (ACQ) score and the severity of the disease was classified based on treatment steps according to GINA 2020. T2 asthma was defined based on any of these criteria using GEMA5.4 guidelines: eosinophils count in blood ≥ 150 µl, allergy (defined by at least one positive prick test or specific IgE), or FeNO ≥ 25 ppb). *Variables from the last 6 months previously to sample collection. Abbreviations: BMI: Body Mass Index; FEV_1: Forced Expiratory Volume in the first second; FVC: Forced Vital Capacity; IgE: Immunoglobulin E; ICS: Inhaled Corticosteroids; N: sample size.

Table S2. Identification of cofounding demographic and clinical variables.

Variable type	Variable		Saliva Bacterial	Saliva Fungal	Saliva Ratio	Pharyngeal Bacterial	Pharyngeal Fungal	Pharyngeal Ratio
			**DNA concentrations[a]**					
			**Saliva**			**Pharyngeal**		
**Variable type**	**Variable**		**Bacterial**	**Fungal**	**Ratio**	**Bacterial**	**Fungal**	**Ratio**
**Demographic**	Age		0.423	**0.003**	**1.78×10^{-04}**	0.400	0.117	0.370
	Sex		**0.025**	0.058	0.608	0.762	0.132	0.387
	Recruiting island		**0.018**	0.197	0.899	0.208	0.439	0.573
	Season		0.157	0.382	0.236	0.410	0.085	0.236
**Clinical**	BMI		0.726	0.752	0.886	0.255	0.074	0.884
	Age of asthma onset		0.901	0.279	0.173	0.509	0.430	0.502
	FEV_1 (% predicted)		0.448	0.899	0.376	0.262	0.899	0.744
	FVC (% predicted)		0.812	0.910	0.637	0.449	0.328	0.860
	FEV_1/FVC (% predicted)		0.259	0.541	0.653	0.512	0.242	0.614
	IgE levels (UI/ml)		0.190	0.889	0.282	0.737	0.080	0.524
	Eosinophil counts (cells/μl)		0.200	0.372	0.426	0.461	0.245	0.451
	Use of antibiotics		0.847	0.601	**0.028**	0.456	0.269	0.190
	Biological therapy		0.082	0.827	0.782	0.541	0.578	0.291
	ICS use		0.069	0.656	0.183	0.123	0.560	0.410
	Systemic corticosteroids use		0.591	0.921	0.640	0.401	0.851	0.790
**Asthma features**	Exacerbations		0.477	0.579	0.918	0.654	0.547	0.553
	Asthma control	Well vs Partially controlled	0.340	0.961	0.676	0.654	0.749	0.552
		Well vs Poorly controlled	0.771	**0.032**	0.319	**0.020**	0.184	0.316
		Partially vs Poorly controlled	0.540	0.290	0.781	**0.033**	0.053	0.572
	Asthma severity	Mild vs Moderate	NA	NA	NA	NA	NA	NA
		Mild vs Severe	0.753	0.072	0.353	0.207	0.155	0.825
		Moderate vs Severe	0.142	0.323	0.522	0.516	0.266	0.561
	T2 asthma		0.628	**9.20×10^{-3}**	0.555	0.812	0.322	**0.047**

[a]Bacterial and fungal DNA concentrations were measured as the copy number of *16S rRNA* or *18S rRNA* genes per nanogram of purified DNA, respectively. The ratio between bacterial and fungal DNA concentrations was also calculated. Associations between variables listed in e-Table 1 with Log transformed microbial DNA concentrations in saliva and pharyngeal samples. Demographic variables were tested with multiple linear regression models adjusted by PCs. Clinical variables were tested with multiple linear regression models adjusted by age, sex, recruiting island, and PCs. Variables related with asthma features were tested with multiple linear regression models adjusted by age, sex, recruiting island, use of antibiotics (last 2 months), and PCs. Statistically significant *p*-values are depicted in boldface (*p*-value < 0.05).

Abbreviations: BMI: Body Mass Index; FVE_1: Forced Expiratory Volume in the first second; FVC: Forced Vital Capacity; IgE: Immunoglobulin E; ICS: Inhaled Corticosteroids; PCs: principal components of genome-wide genotype data; NA: Not Available.

Table S3. Comparations between bacterial, fungal, and ratio of bacterial/fungal DNA concentrations in saliva and pharyngeal samples.

DNA concentrations[a]	Saliva vs. Pharyngeal[b]	FDR[c]
Bacterial	**1.09×10^{-15}** (-0.26 ± 0.03)	**1.63×10^{-15}**
Fungal	**2.55×10^{-18}** (-0.33 ± 0.04)	**7.67×10^{-18}**
Ratio	0.741 (0.02 ± 0.05)	0.741

[a]Bacterial and fungal DNA concentrations were measured as the copy number of *16S rRNA* or *18S rRNA* genes per nanogram of purified DNA, respectively. The ratio between bacterial and fungal DNA concentrations was also calculated. [b]Comparations of Log transformed microbial DNA concentrations between saliva and pharyngeal samples. Multiple linear regression models were adjusted by age, sex, recruiting island, use of antibiotics (last 2 months), and PCs. Statistically significant *p*-values are depicted in boldface (p-value < 0.05), while β values and their corresponding standard deviation are shown in brackets. [c]*p*-values after correction for multiple comparisons (FDR < 0.05).

Abbreviations: PCs: principal components of genome-wide genotype data; FDR: False Discovery Rate.

Table S4. Effect of SDI exposure over bacterial, fungal, and ratio of bacterial/fungal DNA concentrations in saliva and pharyngeal samples.

Biological sample	DNA concentrations[a]	Not-exposed vs. Exposed[b]	FDR[c]
Saliva	Bacterial	**0.022** (0.10 ± 0.04)	**0.022**
	Fungal	**6.32×10^{-5}** (-0.18 ± 0.04)	**1.90×10^{-4}**
	Ratio	**1.02×10^{-3}** (0.22 ± 0.06)	**1.52×10^{-3}**
Pharyngeal	Bacterial	**7.65×10^{-4}** (0.18 ± 0.05)	**1.52×10^{-3}**
	Fungal	**2.68×10^{-3}** (-0.22 ± 0.07)	**3.21×10^{-3}**
	Ratio	**3.36×10^{-5}** (0.37 ± 0.09)	**1.90×10^{-4}**

[a]Bacterial and fungal DNA concentrations were measured as the copy number of *16S rRNA* or *18S rRNA* genes per nanogram of purified DNA, respectively. The ratio between bacterial and fungal DNA concentrations was also calculated.
[b]Comparations of Log transformed microbial DNA concentrations between Not-exposed (0 days of exposition, during the 10 days prior to sampling) and Exposed (exposed to a PM_{10}>50μg/m^3, on any of the 10 days prior to sampling) groups. Multiple linear regression models were adjusted by age, sex, recruiting island, use of antibiotics (last 2 months), and PCs. Statistically significant *p*-values are depicted in boldface (*p*-value < 0.05), while β values and their corresponding standard deviation, are shown in brackets.
[c]*p*-values after correction for multiple comparisons (FDR < 0.05).
Abbreviations: PCs: principal components of genome-wide genotype data; FDR: False Discovery Rate; SDI: Saharan Dust Intrusions.

Table S5. Accumulative effect of SDI exposure over bacterial, fungal, and ratio of bacterial/fungal DNA concentrations in saliva and pharyngeal samples.

Biological sample	DNA concentrations[a]	Not-exposed vs. Low-exposed[b]	FDR[c]	Not-exposed vs. High-exposed[b]	FDR[c]	Low-exposed vs. High-exposed[b]	FDR[c]
Saliva	Bacterial	**0.038** (0.10 ± 0.05)	0.057	**7.11x10^{-3}** (0.16 ± 0.06)	**0.018**	0.977 (0.002 ± 0.06)	0.977
	Fungal	**1.48x10^{-3}** (-0.17 ± 0.05)	**5.88x10^{-3}**	**1.63x10^{-3}** (-0.21 ± 0.07)	**5.88x10^{-3}**	0.177 (-0.07 ± 0.05)	0.228
	Ratio	**1.42x10^{-3}** (0.23 ± 0.07)	**5.88x10^{-3}**	**0.021** (0.21 ± 0.09)	**0.034**	0.778 (-0.03 ± 0.09)	0.875
Pharyngeal	Bacterial	**1.37x10^{-3}** (0.19 ± 0.06)	**5.88x10^{-3}**	**0.015** (0.17 ± 0.07)	**0.026**	0.891 (0.01 ± 0.07)	0.944
	Fungal	**4.39x10^{-3}** (-0.26 ± 0.09)	**0.013**	**9.74x10^{-3}** (-0.27 ± 0.10)	**0.019**	0.598 (0.04 ± 0.08)	0.717
	Ratio	**2.93x10^{-5}** (0.41 ± 0.09)	**5.26x10^{-4}**	**9.29x10^{-3}** (0.30 ± 0.11)	**0.019**	0.165 (-0.16 ± 0.11)	0.228

[a]Bacterial and fungal DNA concentrations were measured as the copy number of *16S rRNA* or *18S rRNA* genes per nanogram of purified DNA, respectively. The ratio between bacterial and fungal DNA concentrations was also calculated.

[b]Comparations of Log transformed microbial DNA concentrations between Not-exposed (0 days of exposition, during the 10 days prior to sampling), Low-exposed (1-3 days of exposition), or High-exposed (4-6 days of exposition) groups. Multiple linear regression models were adjusted by age, sex, recruiting island, use of antibiotics (last 2 months), and PCs. Statistically significant *p*-values are depicted in boldface (p-value < 0.05), while β values and their corresponding standard deviation, are shown in brackets.

[c]*p*-values after correction for multiple comparisons (FDR < 0.05).

Abbreviations: PCs: principal components of genome-wide genotype data; FDR: False Discovery Rate; SDI: Saharan Dust Intrusions.

Table S6. Time-course effect of SDI exposure over bacterial, fungal, and ratio of bacterial/fungal DNA concentrations in saliva and pharyngeal samples.

**Biological sample**	**DNA concentrations[a]**	**Not-exposed vs. Early-exposed[b]**	**FDR[c]**	**Not-exposed vs. Late-exposed[b]**	**FDR[c]**
Saliva	Bacterial	0.268 (0.07 ± 0.07)	0.293	**$8.25x10^{-3}$** (0.15 ± 0.06)	**0.016**
	Fungal	**$1.01x10^{-3}$** (-0.26 ± 0.08)	**$3.02x10^{-3}$**	0.389 (-0.05 ± 0.06)	0.389
	Ratio	**$8.72x10^{-4}$** (0.33 ± 0.10)	**$3.02x10^{-3}$**	**$7.28x10^{-3}$** (0.22 ± 0.08)	**0.016**
Pharyngeal	Bacterial	0.198 (0.11 ± 0.09)	0.238	**$1.00x10^{-4}$** (0.24 ± 0.06)	**$6.01x10^{-4}$**
	Fungal	**0.049** (-0.29 ± 0.15)	0.074	0.122 (-0.15 ± 0.10)	0.162
	Ratio	**0.021** (0.32 ± 0.14)	**0.035**	**$1.23x10^{-5}$** (0.46 ± 0.10)	**$1.47x10^{-4}$**

[a]Bacterial and fungal DNA concentrations were measured as the copy number of *16S rRNA* or *18S rRNA* genes per nanogram of purified DNA, respectively. The ratio between bacterial and fungal DNA concentrations was also calculated.

[b]Comparations of Log transformed microbial DNA concentrations between Not-exposed (0 days of exposition, during the 10 days prior to sampling), Early-exposed (exposed only at days 1-3 before sampling) or Late-exposed (exposed only at days 4-10 prior to sampling) groups. Multiple linear regression models were adjusted by age, sex, recruiting island, use of antibiotics (last 2 months), and PCs. Statistically significant *p*-values are depicted in boldface (p-value < 0.05), while β values and their corresponding standard deviation, are shown in brackets.

[c]*p*-values after correction for multiple comparisons (FDR < 0.05).

Abbreviations: PCs: principal components of genome-wide genotype data; FDR: False Discovery Rate; SDI: Saharan Dust Intrusions.

Table S7. Allele-additive multiple linear regression models to test associations between *SERPINA1* gene variants with bacterial, fungal, and ratio of bacterial/fungal DNA concentrations.

Biological sample	SNV (Allele; frequency)[a]	Bacterial[b]	Fungal[b]	Ratio[b]
Saliva	rs1243163 (A; 0.012)	0.206<br>(0.22±0.17)	0.911<br>(-0.02±0.18)	0.736<br>(-0.10±0.29)
	rs1303 (G; 0.249)	0.697<br>(-0.02±0.05)	0.302<br>(0.05±0.04)	0.739<br>(0.02±0.07)
	rs17580 (A; *Pi*S;* 0.084)	0.523<br>(0.05±0.07)	0.999<br>(-7.9×10^{-5}±0.07)	0.275<br>(0.12±0.11)
	rs2854254 (T; 0.316)	0.217<br>(-0.05±0.04)	0.476<br>(0.03±0.04)	0.072<br>(-0.12±0.07)
	rs28929474 (T; *Pi*Z;* 0.027)	0.766<br>(0.04±0.12)	0.182<br>(0.17±0.12)	0.053<br>(-0.35±0.18)
	rs28931570 (A; 0.005)	0.603<br>(0.20±0.38)	0.369<br>(0.32±0.36)	0.786<br>(0.16±0.58)
	rs6647 (G; 0.219)	0.978<br>(0.001±0.05)	0.469<br>(0.03±0.05)	0.618<br>(-0.04±0.07)
	rs72706301 (A; 0.020)	0.337<br>(-0.14±0.15)	0.987<br>(0.002±0.15)	0.205<br>(-0.28±0.22)
	rs8010121 (G; 0.200)	0.771<br>(-0.02±0.05)	0.394<br>(0.04±0.05)	0.686<br>(0.03±0.08)
Pharyngeal	rs1243163 (A; 0.012)	0.667<br>(0.10±0.23)	0.654<br>(0.10±0.23)	0.892<br>(-0.05±0.37)
	rs1303 (G; 0.249)	0.870<br>(-0.01±0.06)	0.809<br>(0.02±0.07)	0.666<br>(0.04±0.10)
	rs17580 (A; *Pi*S;* 0.084)	0.578<br>(0.05±0.09)	0.347<br>(-0.09±0.10)	0.118<br>(0.21±0.13)
	rs2854254 (T; 0.316)	0.046<br>(-0.11±0.05)	0.053<br>(0.12±0.06)	**2.01×10^{-3}**<br>(-0.28±0.09)
	rs28929474 (T; *Pi*Z;* 0.027)	0.449<br>(-0.15±0.20)	0.059<br>(0.44±0.23)	0.065<br>(-0.54±0.29)
	rs28931570 (A; 0.005)	0.450<br>(-0.24±0.32)	0.984<br>(-0.01±0.46)	0.913<br>(0.07±0.65)
	rs6647 (G; 0.219)	0.543<br>(-0.04±0.06)	0.413<br>(-0.05±0.07)	0.240<br>(-0.11±0.09)
	rs72706301 (A; 0.020)	0.985<br>(0.004±0.20)	0.788<br>(0.06±0.23)	0.346<br>(0.31±0.33)
	rs8010121 (G; 0.200)	0.702<br>(0.03±0.06)	0.914<br>(-0.01±0.07)	0.737<br>(-0.03±0.10)

[a]The copy-number (0, 1, 2 copies) of indicated alleles (minor alleles in the study population referred to the plus-strand of chromosome 14, and their frequencies, are shown) from nine SNVs were tested for associations, using multiple linear regression models adjusted by age, sex, recruiting island, use of antibiotics (last 2 months) and PCs, after Log transformation of DNA concentrations. Statistically significant *p*-values after Bonferroni corrections are depicted in boldface (*p*-value < 0.0056; 0.05/9 comparisons), while β values and their corresponding standard deviation are shown in brackets.

[b]Bacterial and fungal DNA concentrations were measured as the copy number of *16S rRNA* or *18S rRNA* genes per nanogram of purified DNA, respectively. The ratio between bacterial and fungal DNA concentrations was also calculated. Abbreviations: PCs: principal components of genome-wide genotype data; SNV: Single Nucleotide Variant.

Table S8. Allele-additive multiple linear regression models to test associations between SNV rs2854254 with the ratio of bacterial/fungal DNA concentrations in pharyngeal samples stratified by SDI exposure.

	Not-exposed vs. Exposed[b]			rs2854254 genotypes[c]	
	**CC**	**CT**	**TT**	**Not-exposed**	**Exposed**
**Linear regression models[a]**	**$3.75x10^{-5}$** (0.50 ± 0.11)	0.960 (0.01 ± 0.12)	0.927 (-0.13 ± 1.25)	0.331 (-0.14 ± 0.14)	**$2.21x10^{-3}$** (-0.34 ± 0.11)
**FDR[d]**	**$1.12x10^{-4}$**	0.960	0.960	0.331	**$4.42x10^{-3}$**

[a]Allele-additive multiple linear regression models adjusted by age, sex, recruiting island, use of antibiotics (last 2 months), and PCs. Statistically significant *p*-values are depicted in boldface (*p*-value < 0.05) for each linear regression model, after log transformation of DNA concentration's ratio, while the β values and their corresponding standard deviation, are shown in brackets.

[b]Patients were distributed considering their SDI exposure status and, inside each exposure-group, the pharyngeal ratio of bacterial/fungal DNA concentrations was compared between rs2854254 genotypes.

[c]Patients were distributed considering their genotype for the SNV rs2854254 and, inside each genotype-group, the pharyngeal ratio of bacterial/fungal DNA concentrations was compared between Exposed (exposure to a PM_{10}>50μg/m^3, on any of the 10 days prior to sampling) and Not-exposed (0 days of exposition, during the 10 days prior to sampling individuals) groups.

[d]*p*-values after correction for multiple comparisons (FDR < 0.05).

Abbreviations: PCs: principal components of genome-wide genotype data; FDR: False Discovery Rate; SDI: Saharan Dust Intrusions; SNV: Single Nucleotide Variant.

Table S9. PM_{10} concentration levels between the 10 days prior to sampling for each asthmatic patient.

ID[a]	1 day	2 day	3 day	4 day	5 day	6 day	7 day	8 day	9 day	10 day	Cumulative[b]	Minimum[c]	Maximum[d]	Average[e]	SDI exposure[f]
0001	17.819	10.891	16.672	22.35	15.544	15.965	12.012	10.785	6.872	7.739	136.65	6.87	22.35	13.66	0
0002	18.126	16.662	16.765	14.282	11.959	16.185	17.584	17.819	10.891	16.672	156.95	10.89	18.13	15.69	0
0003	19.378	18.126	16.662	16.765	14.282	11.959	16.185	17.584	17.819	10.891	159.65	10.89	19.38	15.97	0
0004	15.127	11.208	11.91	19.378	18.126	16.662	16.765	14.282	11.959	16.185	151.60	11.21	19.38	15.16	0
0005	15.127	11.208	11.91	19.378	18.126	16.662	16.765	14.282	11.959	16.185	151.60	11.21	19.38	15.16	0
0006	9.105	12.488	13.417	16.396	11.834	14.276	14.159	12.277	16.541	17.764	138.26	9.11	17.76	13.83	0
0007	6.802	7.449	8.321	8.497	12.614	13.135	12.900	8.781	8.161	12.992	99.65	6.80	13.14	9.97	0
0008	19.964	53.244	38.624	24.328	27.023	45.352	36.507	34.686	40.109	32.372	352.21	19.96	53.24	35.22	1
0010	17.885	20.219	18.628	15.585	11.302	11.301	15.337	19.168	20.501	22.169	172.10	11.30	22.17	17.21	0
0011	22.831	22.456	26.266	27.647	26.585	29.081	30.029	26.205	29.298	34.96	275.36	22.46	34.96	27.54	0
0012	16.311	27.67	71.843	57.109	12.954	11.941	12.679	19.562	19.584	22.709	272.36	11.94	71.84	27.24	2
0013	12.277	16.541	17.764	16.107	14.752	12.846	9.681	10.464	11.416	12.619	134.47	9.68	17.76	13.45	0
0014	14.153	17.956	15.073	29.023	100.47	212.08	39.788	17.937	16.718	10.044	473.24	10.04	212.08	47.32	2
0015	27.647	26.585	29.081	30.029	26.205	29.298	34.96	29.511	34.411	42.147	309.87	26.21	42.15	30.99	0
0016	11.903	15.937	23.082	24.47	15.205	16.461	18.43	17.242	15.61	11.583	169.92	11.58	24.47	16.99	0
0017	11.903	15.937	23.082	24.47	15.205	16.461	18.43	17.242	15.61	11.583	169.92	11.58	24.47	16.99	0
0018	71.843	57.109	12.954	11.941	12.679	19.562	19.584	22.709	30.993	15.296	274.67	11.94	71.84	27.47	2
0019	37.471	26.015	32.502	69.027	93.654	96.785	227.59	61.61	23.225	17.772	685.65	17.77	227.59	68.57	5
0020	37.471	26.015	32.502	69.027	93.654	96.785	227.59	61.61	23.225	17.772	685.65	17.77	227.59	68.57	5
0021	50.955	25.338	32.782	37.471	26.015	32.502	69.027	93.654	96.785	227.59	692.12	25.34	227.59	69.21	5
0022	32.782	37.471	26.015	32.502	69.027	93.654	96.785	227.59	61.61	23.225	700.66	23.23	227.59	70.07	5
0023	37.471	26.015	32.502	69.027	93.654	96.785	227.59	61.61	23.225	17.772	685.65	17.77	227.59	68.57	5
0024	32.782	37.471	26.015	32.502	69.027	93.654	96.785	227.59	61.61	23.225	700.66	23.23	227.59	70.07	5
0025	50.955	25.338	32.782	37.471	26.015	32.502	69.027	93.654	96.785	227.59	692.12	25.34	227.59	69.21	5
0026	25.338	32.782	37.471	26.015	32.502	69.027	93.654	96.785	227.59	61.61	702.78	25.34	227.59	70.28	5
0027	32.782	37.471	26.015	32.502	69.027	93.654	96.785	227.59	61.61	23.225	700.66	23.23	227.59	70.07	5
0028	32.782	37.471	26.015	32.502	69.027	93.654	96.785	227.59	61.61	23.225	700.66	23.23	227.59	70.07	5
0029	32.782	37.471	26.015	32.502	69.027	93.654	96.785	227.59	61.61	23.225	700.66	23.23	227.59	70.07	5
0030	32.999	66.707	15.661	24.827	50.955	25.338	32.782	37.471	26.015	32.502	345.26	15.66	66.71	34.53	2
1001	11.903	15.937	23.082	24.47	15.205	16.461	18.43	17.242	15.61	11.583	169.92	11.58	24.47	16.99	0
1002	14.927	16.255	13.293	16.319	18.208	24.469	14.347	10.463	8.727	15.228	152.24	8.73	24.47	15.22	0
1003	11.743	14.01	10.958	8.935	10.064	9.806	12.02	15.535	14.905	23.899	131.88	8.94	23.90	13.19	0
1004	16.609	20.35	23.977	21.865	14.997	16.665	12.97	19.127	18.957	14.189	179.71	12.97	23.98	17.97	0
1005	12.671	16.609	20.35	23.977	21.865	14.997	16.665	12.97	19.127	18.957	178.19	12.67	23.98	17.82	0
1006	11.788	12.405	15.388	19.988	13.809	12.671	16.609	20.35	23.977	21.865	168.85	11.79	23.98	16.89	0
1007	10.144	9.105	12.488	13.417	16.396	11.834	14.276	14.159	12.277	16.541	130.64	9.11	16.54	13.06	0
1008	12.86	14.401	19.781	25.238	27.22	22.11	18.181	17.200	18.684	10.755	186.43	10.76	27.22	18.64	0
1009	16.983	19.071	19.134	15.114	14.978	12.112	11.631	12.860	14.401	19.781	156.07	11.63	19.78	15.61	0
1010	17.983	9.353	12.298	11.32	13.32	13.636	17.406	21.052	22.831	22.456	161.66	9.35	22.83	16.17	0

1011	27.67	71.843	57.109	12.954	11.941	12.679	19.562	19.584	22.709	30.993	287.04	11.94	71.84	28.70	2
1012	16.311	27.67	71.843	57.109	12.954	11.941	12.679	19.562	19.584	22.709	272.36	11.94	71.84	27.24	2
1013	10.645	10.911	12.791	13.093	17.36	12.123	12.164	12.565	11.302	8.516	121.47	8.52	17.36	12.15	0
1201	8.497	12.614	13.135	12.900	8.781	8.161	12.992	9.699	16.311	27.67	130.76	8.16	27.67	13.08	0
1203	12.126	11.073	11.464	12.179	14.627	14.714	13.119	11.79	13.71	16.619	131.42	11.07	16.62	13.14	0
1204	25.338	32.782	37.471	26.015	32.502	69.027	93.654	96.785	227.59	61.61	702.78	25.34	227.59	70.28	5
1206	17.471	18.949	19.001	19.048	18.77	11.635	8.368	11.108	17.906	20.163	162.42	8.37	20.16	16.24	0
1207	17.471	18.949	19.001	19.048	18.77	11.635	8.368	11.108	17.906	20.163	162.42	8.37	20.16	16.24	0
1401	13.415	13.241	10.72	15.006	21.374	23.751	27.093	18.644	15.64	12.508	171.39	10.72	27.09	17.14	0
1402	12.274	12.813	13.415	13.241	10.72	15.006	21.374	23.751	27.093	18.644	168.33	10.72	27.09	16.83	0
1403	12.813	13.415	13.241	10.72	15.006	21.374	23.751	27.093	18.644	15.64	171.70	10.72	27.09	17.17	0
1404	26.528	16.623	10.725	12.743	12.274	12.813	13.415	13.241	10.72	15.006	144.09	10.72	26.53	14.41	0
1405	26.800	27.135	23.308	22.96	16.187	19.132	19.129	19.964	53.244	38.624	266.48	16.19	53.24	26.65	1
1406	12.635	17.11	19.435	17.471	18.949	19.001	19.048	18.77	11.635	8.368	162.42	8.37	19.44	16.24	0
1407	19.892	17.281	13.962	22.52	27.586	26.04	26.501	18.456	12.635	17.11	201.98	12.64	27.59	20.20	0
1408	11.108	17.906	20.163	19.226	14.825	12.902	15.134	12.875	13.215	12.42	149.77	11.11	20.16	14.98	0
1409	11.108	17.906	20.163	19.226	14.825	12.902	15.134	12.875	13.215	12.42	149.77	11.11	20.16	14.98	0
1410	12.42	14.299	12.126	11.073	11.464	12.179	14.627	14.714	13.119	11.79	127.81	11.07	14.71	12.78	0
1411	21.237	15.648	13.096	15.855	18.776	24.874	31.656	25.548	22.831	20.846	210.37	13.10	31.66	21.04	0
1412	12.274	12.813	13.415	13.241	10.72	15.006	21.374	23.751	27.093	18.644	168.33	10.72	27.09	16.83	0
1413	17.471	18.949	19.001	19.048	18.77	11.635	8.368	11.108	17.906	20.163	162.42	8.37	20.16	16.24	0
1414	11.108	17.906	20.163	19.226	14.825	12.902	15.134	12.875	13.215	12.42	149.77	11.11	20.16	14.98	0
1415	18.77	11.635	8.368	11.108	17.906	20.163	19.226	14.825	12.902	15.134	150.04	8.37	20.16	15.00	0
1416	19.226	14.825	12.902	15.134	12.875	13.215	12.42	14.299	12.126	11.073	138.10	11.07	19.23	13.81	0
1417	11.108	17.906	20.163	19.226	14.825	12.902	15.134	12.875	13.215	12.42	149.77	11.11	20.16	14.98	0
1418	12.875	13.215	12.42	14.299	12.126	11.073	11.464	12.179	14.627	14.714	128.99	11.07	14.71	12.90	0
1419	12.42	14.299	12.126	11.073	11.464	12.179	14.627	14.714	13.119	11.79	127.81	11.07	14.71	12.78	0
1420	12.126	11.073	11.464	12.179	14.627	14.714	13.119	11.79	13.71	16.619	131.42	11.07	16.62	13.14	0
1421	12.133	12.098	12.63	11.486	16.011	14.738	23.069	30.939	24.434	31.411	188.95	11.49	31.41	18.89	0
1422	12.133	12.098	12.63	11.486	16.011	14.738	23.069	30.939	24.434	31.411	188.95	11.49	31.41	18.89	0
1423	11.486	16.011	14.738	23.069	30.939	24.434	31.411	31.503	21.296	18.738	223.63	11.49	31.50	22.36	0
1424	19.964	53.244	38.624	24.328	27.023	45.352	36.507	34.686	40.109	32.372	352.21	19.96	53.24	35.22	1
1425	30.939	24.434	31.411	31.503	21.296	18.738	32.812	24.551	43.961	47.804	307.45	18.74	47.80	30.74	0
1426	16.187	19.132	19.129	19.964	53.244	38.624	24.328	27.023	45.352	36.507	299.49	16.19	53.24	29.95	1
1428	53.244	38.624	24.328	27.023	45.352	36.507	34.686	40.109	32.372	23.974	356.22	23.97	53.24	35.62	1
1429	16.187	19.132	19.129	19.964	53.244	38.624	24.328	27.023	45.352	36.507	299.49	16.19	53.24	29.95	1
1430	19.129	19.964	53.244	38.624	24.328	27.023	45.352	36.507	34.686	40.109	338.97	19.13	53.24	33.90	1
1431	19.168	20.501	22.169	26.581	21.381	21.786	24.041	27.299	21.706	25.101	229.73	19.17	27.30	22.97	0
1432	19.168	20.501	22.169	26.581	21.381	21.786	24.041	27.299	21.706	25.101	229.73	19.17	27.30	22.97	0
1433	43.961	47.804	42.32	27.177	20.932	19.848	17.885	20.219	18.628	15.585	274.36	15.59	47.80	27.44	0
1434	17.069	25.771	19.631	26.8	27.135	23.308	22.96	16.187	19.132	19.129	217.12	16.19	27.14	21.71	0

1435	21.296	18.738	32.812	24.551	43.961	47.804	42.32	27.177	20.932	19.848	299.44	18.74	47.80	29.94	0
1436	20.219	18.628	15.585	11.302	11.301	15.337	19.168	20.501	22.169	26.581	180.79	11.30	26.58	18.08	0
1437	43.961	47.804	42.32	27.177	20.932	19.848	17.885	20.219	18.628	15.585	274.36	15.59	47.80	27.44	0
1438	18.628	15.585	11.302	11.301	15.337	19.168	20.501	22.169	26.581	21.381	181.95	11.30	26.58	18.20	0
1439	19.631	26.8	27.135	23.308	22.96	16.187	19.132	19.129	19.964	53.244	247.49	16.19	53.24	24.75	1
1440	17.069	25.771	19.631	26.8	27.135	23.308	22.96	16.187	19.132	19.129	217.12	16.19	27.14	21.71	0
1441	40.831	35.414	22.874	15.858	9.969	12.23	15.949	12.625	12.782	17.9	196.43	9.97	40.83	19.64	0
1442	87.14	37.5	44.717	19.78	15.65	18.268	18.019	21.162	19.382	12.488	294.11	12.49	87.14	29.41	1
1443	18.019	21.162	19.382	12.488	17.528	24.857	41.038	89.078	49.123	21.744	314.42	12.49	89.08	31.44	1
1444	22.874	15.858	9.969	12.23	15.949	12.625	12.782	17.9	19.44	21.092	160.72	9.97	22.87	16.07	0
1445	17.9	19.44	21.092	20.881	22.446	52.699	87.14	37.5	44.717	19.78	343.60	17.90	87.14	34.36	2
1446	265.36	42.881	20.007	29.024	30.978	40.831	35.414	22.874	15.858	9.969	513.20	9.97	265.36	51.32	1
1447	13.642	18.086	18.778	17.069	25.771	19.631	26.8	27.135	23.308	22.96	213.18	13.64	27.14	21.32	0
1448	23.781	18.188	17.009	22.357	23.82	24.151	23.935	18.006	19.117	18.678	209.04	17.01	24.15	20.90	0
1449	18.019	21.162	19.382	12.488	17.528	24.857	41.038	89.078	49.123	21.744	314.42	12.49	89.08	31.44	1
1450	23.781	18.188	17.009	22.357	23.82	24.151	23.935	18.006	19.117	18.678	209.04	17.01	24.15	20.90	0
1454	265.36	42.881	20.007	29.024	30.978	40.831	35.414	22.874	15.858	9.969	513.20	9.97	265.36	51.32	1
1455	39.845	240.98	265.36	42.881	20.007	29.024	30.978	40.831	35.414	22.874	768.19	20.01	265.36	76.82	2
1456	25.564	57.192	37.547	20.785	24.691	19.122	33.52	134.01	555.5	713.21	1621.15	19.12	713.21	162.11	4
1460	54.884	28.179	20.391	98.029	70.235	39.845	240.98	265.36	42.881	20.007	880.79	20.01	265.36	88.08	5
1461	240.98	265.36	42.881	20.007	29.024	30.978	40.831	35.414	22.874	15.858	744.20	15.86	265.36	74.42	2
1462	24.929	27.245	28.3	29.45	19.113	36.662	63.497	66.862	69.246	54.884	420.19	19.11	69.25	42.02	4
1463	27.245	28.3	29.45	19.113	36.662	63.497	66.862	69.246	54.884	28.179	423.44	19.11	69.25	42.34	4
1464	23.619	25.918	25.564	57.192	37.547	20.785	24.691	19.122	33.52	134.01	401.97	19.12	134.01	40.20	2
1466	46.686	38.294	39.556	26.969	20.054	25.424	26.063	23.711	19.079	24.351	290.19	19.08	46.69	29.02	0
1501	8.497	12.614	13.135	12.9	8.781	8.161	12.992	9.699	16.311	27.67	130.76	8.16	27.67	13.08	0
1502	12.614	13.135	12.9	8.781	8.161	12.992	9.699	16.311	27.67	71.843	194.11	8.16	71.84	19.41	1
1503	67.506	61.727	72.188	108.08	128.63	78.074	15.399	10.224	13.96	32.999	588.79	10.22	128.63	58.88	6
1504	12.614	13.135	12.9	8.781	8.161	12.992	9.699	16.311	27.67	71.843	194.11	8.16	71.84	19.41	1
1505	8.497	12.614	13.135	12.9	8.781	8.161	12.992	9.699	16.311	27.67	130.76	8.16	27.67	13.08	0
1506	12.614	13.135	12.9	8.781	8.161	12.992	9.699	16.311	27.67	71.843	194.11	8.16	71.84	19.41	1
1507	6.802	7.449	8.321	8.497	12.614	13.135	12.9	8.781	8.161	12.992	99.65	6.80	13.14	9.97	0
1508	108.08	128.63	78.074	15.399	10.224	13.96	32.999	66.707	15.661	24.827	494.57	10.22	128.63	49.46	4
1509	13.135	12.9	8.781	8.161	12.992	9.699	16.311	27.67	71.843	57.109	238.60	8.16	71.84	23.86	2
1510	13.135	12.9	8.781	8.161	12.992	9.699	16.311	27.67	71.843	57.109	238.60	8.16	71.84	23.86	2
1511	13.451	6.802	7.449	8.321	8.497	12.614	13.135	12.9	8.781	8.161	100.11	6.80	13.45	10.01	0
1512	13.135	12.9	8.781	8.161	12.992	9.699	16.311	27.67	71.843	57.109	238.60	8.16	71.84	23.86	2
1513	12.614	13.135	12.9	8.781	8.161	12.992	9.699	16.311	27.67	71.843	194.11	8.16	71.84	19.41	1
1514	8.497	12.614	13.135	12.9	8.781	8.161	12.992	9.699	16.311	27.67	130.76	8.16	27.67	13.08	0
1515	6.802	7.449	8.321	8.497	12.614	13.135	12.9	8.781	8.161	12.992	99.65	6.80	13.14	9.97	0
1516	93.654	96.785	227.59	61.61	23.225	17.772	9.779	8.46	8.417	12.664	559.96	8.42	227.59	56.00	4

1517	227.59	61.61	23.225	17.772	9.779	8.46	8.417	12.664	13.451	6.802	389.77	6.80	227.59	38.98	2
1518	227.59	61.61	23.225	17.772	9.779	8.46	8.417	12.664	13.451	6.802	389.77	6.80	227.59	38.98	2
1519	96.785	227.59	61.61	23.225	17.772	9.779	8.46	8.417	12.664	13.451	479.75	8.42	227.59	47.98	3
1520	96.785	227.59	61.61	23.225	17.772	9.779	8.46	8.417	12.664	13.451	479.75	8.42	227.59	47.98	3
1521	93.654	96.785	227.59	61.61	23.225	17.772	9.779	8.46	8.417	12.664	559.96	8.42	227.59	56.00	4
1522	61.61	23.225	17.772	9.779	8.46	8.417	12.664	13.451	6.802	7.449	169.63	6.80	61.61	16.96	1
1523	96.785	227.59	61.61	23.225	17.772	9.779	8.46	8.417	12.664	13.451	479.75	8.42	227.59	47.98	3
1524	61.61	23.225	17.772	9.779	8.46	8.417	12.664	13.451	6.802	7.449	169.63	6.80	61.61	16.96	1
1525	12.664	13.451	6.802	7.449	8.321	8.497	12.614	13.135	12.9	8.781	104.61	6.80	13.45	10.46	0
1526	12.664	13.451	6.802	7.449	8.321	8.497	12.614	13.135	12.9	8.781	104.61	6.80	13.45	10.46	0
1527	27.458	13.317	10.21	22.955	25.365	19.904	94.053	176.34	155.96	61.464	607.02	10.21	176.34	60.70	4
1528	8.46	8.417	12.664	13.451	6.802	7.449	8.321	8.497	12.614	13.135	99.81	6.80	13.45	9.98	0
1529	96.785	227.59	61.61	23.225	17.772	9.779	8.46	8.417	12.664	13.451	479.75	8.42	227.59	47.98	3
1530	96.785	227.59	61.61	23.225	17.772	9.779	8.46	8.417	12.664	13.451	479.75	8.42	227.59	47.98	3
1531	12.664	13.451	6.802	7.449	8.321	8.497	12.614	13.135	12.9	8.781	104.61	6.80	13.45	10.46	0
1532	93.654	96.785	227.59	61.61	23.225	17.772	9.779	8.46	8.417	12.664	559.96	8.42	227.59	56.00	4
1533	96.785	227.59	61.61	23.225	17.772	9.779	8.46	8.417	12.664	13.451	479.75	8.42	227.59	47.98	3
1534	12.664	13.451	6.802	7.449	8.321	8.497	12.614	13.135	12.9	8.781	104.61	6.80	13.45	10.46	0
1535	227.59	61.61	23.225	17.772	9.779	8.46	8.417	12.664	13.451	6.802	389.77	6.80	227.59	38.98	2
1536	93.654	96.785	227.59	61.61	23.225	17.772	9.779	8.46	8.417	12.664	559.96	8.42	227.59	56.00	4
1537	26.015	32.502	69.027	93.654	96.785	227.59	61.61	23.225	17.772	9.779	657.96	9.78	227.59	65.80	5
1538	17.474	30.463	27.458	13.317	10.21	22.955	25.365	19.904	94.053	176.34	437.54	10.21	176.34	43.75	2
1539	227.59	61.61	23.225	17.772	9.779	8.46	8.417	12.664	13.451	6.802	389.77	6.80	227.59	38.98	2
1540	17.474	30.463	27.458	13.317	10.21	22.955	25.365	19.904	94.053	176.34	437.54	10.21	176.34	43.75	2
1541	15.399	10.224	13.96	32.999	66.707	15.661	24.827	50.955	25.338	32.782	288.85	10.22	66.71	28.89	2
1542	15.806	8.118	9.682	8.91	11.657	12.357	32.779	67.506	61.727	72.188	300.73	8.12	72.19	30.07	3
1543	13.96	32.999	66.707	15.661	24.827	50.955	25.338	32.782	37.471	26.015	326.72	13.96	66.71	32.67	2
1544	13.96	32.999	66.707	15.661	24.827	50.955	25.338	32.782	37.471	26.015	326.72	13.96	66.71	32.67	2
1545	72.188	108.08	128.63	78.074	15.399	10.224	13.96	32.999	66.707	15.661	541.93	10.22	128.63	54.19	5
1546	15.399	10.224	13.96	32.999	66.707	15.661	24.827	50.955	25.338	32.782	288.85	10.22	66.71	28.89	2
1547	15.399	10.224	13.96	32.999	66.707	15.661	24.827	50.955	25.338	32.782	288.85	10.22	66.71	28.89	2
1548	15.399	10.224	13.96	32.999	66.707	15.661	24.827	50.955	25.338	32.782	288.85	10.22	66.71	28.89	2
1549	15.806	8.118	9.682	8.91	11.657	12.357	32.779	67.506	61.727	72.188	300.73	8.12	72.19	30.07	3
1550	9.682	8.91	11.657	12.357	32.779	67.506	61.727	72.188	108.08	128.63	513.52	8.91	128.63	51.35	5
1551	108.08	128.63	78.074	15.399	10.224	13.96	32.999	66.707	15.661	24.827	494.57	10.22	128.63	49.46	4
1552	15.399	10.224	13.96	32.999	66.707	15.661	24.827	50.955	25.338	32.782	288.85	10.22	66.71	28.89	2
1553	72.188	108.08	128.63	78.074	15.399	10.224	13.96	32.999	66.707	15.661	541.93	10.22	128.63	54.19	5
1554	9.682	8.91	11.657	12.357	32.779	67.506	61.727	72.188	108.08	128.63	513.52	8.91	128.63	51.35	5
1555	61.727	72.188	108.08	128.63	78.074	15.399	10.224	13.96	32.999	66.707	587.99	10.22	128.63	58.80	6
1556	42.014	35.237	28.538	18.645	15.216	15.806	8.118	9.682	8.91	11.657	193.82	8.12	42.01	19.38	0
1557	9.682	8.91	11.657	12.357	32.779	67.506	61.727	72.188	108.08	128.63	513.52	8.91	128.63	51.35	5

1558	42.014	35.237	28.538	18.645	15.216	15.806	8.118	9.682	8.91	11.657	193.82	8.12	42.01	19.38	0
1559	15.399	10.224	13.96	32.999	66.707	15.661	24.827	50.955	25.338	32.782	288.85	10.22	66.71	28.89	2
1560	32.999	66.707	15.661	24.827	50.955	25.338	32.782	37.471	26.015	32.502	345.26	15.66	66.71	34.53	2
1561	72.188	108.08	128.63	78.074	15.399	10.224	13.96	32.999	66.707	15.661	541.93	10.22	128.63	54.19	5
1562	9.682	8.91	11.657	12.357	32.779	67.506	61.727	72.188	108.08	128.63	513.52	8.91	128.63	51.35	5
1563	13.96	32.999	66.707	15.661	24.827	50.955	25.338	32.782	37.471	26.015	326.72	13.96	66.71	32.67	2
1564	42.014	35.237	28.538	18.645	15.216	15.806	8.118	9.682	8.91	11.657	193.82	8.12	42.01	19.38	0
1565	32.999	66.707	15.661	24.827	50.955	25.338	32.782	37.471	26.015	32.502	345.26	15.66	66.71	34.53	2
1566	15.399	10.224	13.96	32.999	66.707	15.661	24.827	50.955	25.338	32.782	288.85	10.22	66.71	28.89	2
1567	72.188	108.08	128.63	78.074	15.399	10.224	13.96	32.999	66.707	15.661	541.93	10.22	128.63	54.19	5
1568	15.806	8.118	9.682	8.91	11.657	12.357	32.779	67.506	61.727	72.188	300.73	8.12	72.19	30.07	3
1569	42.014	35.237	28.538	18.645	15.216	15.806	8.118	9.682	8.91	11.657	193.82	8.12	42.01	19.38	0
1570	61.727	72.188	108.08	128.63	78.074	15.399	10.224	13.96	32.999	66.707	587.99	10.22	128.63	58.80	6
1571	67.506	61.727	72.188	108.08	128.63	78.074	15.399	10.224	13.96	32.999	588.79	10.22	128.63	58.88	6
1572	67.506	61.727	72.188	108.08	128.63	78.074	15.399	10.224	13.96	32.999	588.79	10.22	128.63	58.88	6
1573	72.188	108.08	128.63	78.074	15.399	10.224	13.96	32.999	66.707	15.661	541.93	10.22	128.63	54.19	5
1574	72.188	108.08	128.63	78.074	15.399	10.224	13.96	32.999	66.707	15.661	541.93	10.22	128.63	54.19	5
1575	72.188	108.08	128.63	78.074	15.399	10.224	13.96	32.999	66.707	15.661	541.93	10.22	128.63	54.19	5
1576	61.727	72.188	108.08	128.63	78.074	15.399	10.224	13.96	32.999	66.707	587.99	10.22	128.63	58.80	6
1577	72.188	108.08	128.63	78.074	15.399	10.224	13.96	32.999	66.707	15.661	541.93	10.22	128.63	54.19	5
1578	67.506	61.727	72.188	108.08	128.63	78.074	15.399	10.224	13.96	32.999	588.79	10.22	128.63	58.88	6
1579	15.399	10.224	13.96	32.999	66.707	15.661	24.827	50.955	25.338	32.782	288.85	10.22	66.71	28.89	2
1580	108.08	128.63	78.074	15.399	10.224	13.96	32.999	66.707	15.661	24.827	494.57	10.22	128.63	49.46	4
1581	108.08	128.63	78.074	15.399	10.224	13.96	32.999	66.707	15.661	24.827	494.57	10.22	128.63	49.46	4
1582	15.399	10.224	13.96	32.999	66.707	15.661	24.827	50.955	25.338	32.782	288.85	10.22	66.71	28.89	2
1583	9.682	8.91	11.657	12.357	32.779	67.506	61.727	72.188	108.08	128.63	513.52	8.91	128.63	51.35	5
1584	21.497	31.85	42.014	35.237	28.538	18.645	15.216	15.806	8.118	9.682	226.60	8.12	42.01	22.66	0
1585	42.014	35.237	28.538	18.645	15.216	15.806	8.118	9.682	8.91	11.657	193.82	8.12	42.01	19.38	0
1586	31.85	42.014	35.237	28.538	18.645	15.216	15.806	8.118	9.682	8.91	214.02	8.12	42.01	21.40	0
1587	8.118	9.682	8.91	11.657	12.357	32.779	67.506	61.727	72.188	108.08	393.01	8.12	108.08	39.30	4
1588	10.224	13.96	32.999	66.707	15.661	24.827	50.955	25.338	32.782	37.471	310.92	10.22	66.71	31.09	2
1589	44.668	37.19	33.235	32.336	25.326	21.725	28.497	29.563	33.888	31.364	317.79	21.73	44.67	31.78	0
1590	54.884	28.179	20.391	98.029	70.235	39.845	240.98	265.36	42.881	20.007	880.79	20.01	265.36	88.08	5
1593	44.668	37.19	33.235	32.336	25.326	21.725	28.497	29.563	33.888	31.364	317.79	21.73	44.67	31.78	0
1595	28.497	29.563	33.888	31.364	27.049	28.249	28.656	26.528	16.623	10.725	261.14	10.73	33.89	26.11	0
1596	19.892	17.281	13.962	22.52	27.586	26.04	26.501	18.456	12.635	17.11	201.98	12.64	27.59	20.20	0
1598	14.022	13.719	17.213	22.005	19.268	11.999	11.039	15.102	18.81	14.647	157.82	11.04	22.01	15.78	0
1600	29.563	33.888	31.364	27.049	28.249	28.656	26.528	16.623	10.725	12.743	245.39	10.73	33.89	24.54	0
1601	30.414	44.668	37.19	33.235	32.336	25.326	21.725	28.497	29.563	33.888	316.84	21.73	44.67	31.68	0
1602	15.139	13.144	19.19	21.737	19.389	21.281	23.763	24.456	23.811	15.501	197.41	13.14	24.46	19.74	0
1604	14.686	16.468	19.254	23.093	29.623	25.969	27.278	34.209	47.087	46.686	284.35	14.69	47.09	28.44	0

1606	39.173	53.031	49.405	29.758	30.703	23.619	25.918	25.564	57.192	37.547	371.91	23.62	57.19	37.19	2
1613	31.955	36.283	32.333	34.622	36.401	31.002	33.716	41.902	56.382	57.287	391.88	31.00	57.29	39.19	2
1614	21.725	28.497	29.563	33.888	31.364	27.049	28.249	28.656	26.528	16.623	272.14	16.62	33.89	27.21	0
1615	35.414	22.874	15.858	9.969	12.23	15.949	12.625	12.782	17.9	19.44	175.04	9.97	35.41	17.50	0
1617	44.668	37.19	33.235	32.336	25.326	21.725	28.497	29.563	33.888	31.364	317.79	21.73	44.67	31.78	0
1618	18.949	19.001	19.048	18.77	11.635	8.368	11.108	17.906	20.163	19.226	164.17	8.37	20.16	16.42	0
1619	18.949	19.001	19.048	18.77	11.635	8.368	11.108	17.906	20.163	19.226	164.17	8.37	20.16	16.42	0
1621	32.336	25.326	21.725	28.497	29.563	33.888	31.364	27.049	28.249	28.656	286.65	21.73	33.89	28.67	0

[a]Identification number for each patient.

[b]Total sum of the PM_{10} concentrations every day prior to sampling.

[c]Minimum value of PM_{10} concentration.

[d]Maximum value of PM_{10} concentration.

[e]Average of PM_{10} concentration between the 10 days prior to sampling.

[f]Total number of days with a PM_{10} concentration ≥ 50μg/m^3.

Supplementary Figure

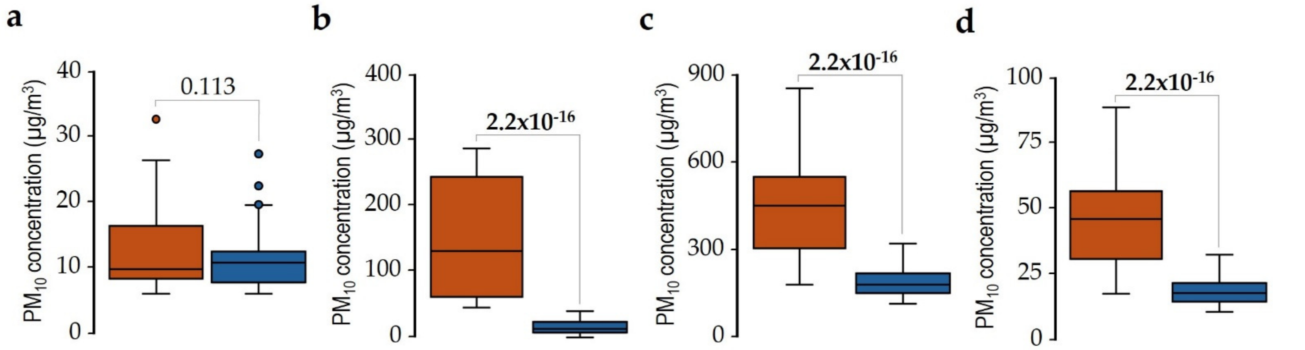

Figure S1. Distribution of PM_{10} concentrations in individuals Exposed (orange) and Not-exposed (blue) to SDI during the 10 days prior to sampling. (**a**) Minimum, (**b**) maximum, (**c**) cumulative, and (**d**) average of daily PM_{10} concentrations 10 days prior to sample collection. The Mann-Whitney U test and t-Student test were used depending on data normality. Significant *p*-values (< 0.05) are highlighted in bold.
